# Incidence of post-induction hypotension following emergency rapid sequence induction with ketamine: a systematic review and meta-analysis

**DOI:** 10.1186/s13049-025-01374-7

**Published:** 2025-05-01

**Authors:** Pedro Vila de Mucha, Stephen Thomas

**Affiliations:** 1 Blizard Institute, Queen Mary University of London, London, UK; 2 Department of Emergency Medicine, Beth Israel Deaconess Medical Center and Harvard Medical School, Boston, MA USA

**Keywords:** Ketamine, Rapid sequence induction, Hypotension

## Abstract

**Introduction:**

Rapid sequence induction (RSI) is a potentially-life saving intervention in critically ill patients. An important adverse effect of this procedure is post-induction hypotension (PIH), which is associated with worsened patient outcomes. Choice of induction agent can affect incidence of PIH, although the optimal drug has yet to be determined. Ketamine is postulated to reduce PIH incidence in emergency RSI when used instead of alternative agents.

**Aims:**

This systematic review and meta-analysis aims to evaluate the effect on PIH incidence of inducing anaesthesia with ketamine during emergency RSI.

**Methods:**

A systematic search was conducted to identify a sample of studies fulfilling criteria for population (emergency RSI), intervention (ketamine), comparator (any alternative induction agent) and outcome (PIH). No single definition of PIH was required for eligibility. A random-effects model was used to produce a pooled effect size estimate from the extracted data. The study question was also tested in pre-specified subgroups, including by specific comparator induction agent and by indication for RSI (medical vs trauma).

**Results:**

27 studies, including 6 randomised controlled trials, were eligible for inclusion, with total n = 31,956. There was considerable methodological heterogeneity. The pooled estimate of odds ratio (OR) of PIH when ketamine is used for emergency RSI is 1.10, with 95% confidence interval 0.78–1.56. Excluding data from the 6 studies (1 randomised and 5 observational) at greater risk of bias, the pooled OR is 0.99 (0.69–1.43). There was no significant difference between ketamine and comparators in any subgroup, although significance was approached when comparing ketamine to etomidate, with OR 1.38 (0.99–1.94) and *p* = 0.058.

**Conclusions:**

Choice of ketamine to carry out emergency RSI did not affect the incidence of PIH incidence in this diverse sample of studies. Given the breadth of inclusion criteria, applicability of this result is not necessarily universal. It is likely that optimal choice of induction agent varies according to specific circumstances in a manner as yet incompletely understood.

**Supplementary Information:**

The online version contains supplementary material available at 10.1186/s13049-025-01374-7.

## Introduction

Rapid sequence induction (RSI) of anaesthesia to facilitate emergent endotracheal intubation is a potentially life-saving intervention in the management of the critically ill. Indications range from requirement for airway protection or invasive ventilation to obtundation from haemodynamic shock. It is recognised as a high-risk procedure with numerous potential adverse effects, including post-induction hypotension (PIH) [1, 2].

PIH following RSI has been reported to occur with incidence which is variable but potentially in excess of 20% [2, 3]. It is associated with increased mortality [2, 3].

Ketamine is postulated to induce anaesthesia with lower risk of cardiovascular instability; its preferential use potentially reduces incidence of PIH. It exerts its clinical effects via antagonism of the NMDA receptor in the central nervous system, inducing a state of dissociative anaesthesia in which patients can safely receive neuromuscular blockade for endotracheal intubation [4–7]. It has been adopted as the induction agent of choice by many UK HEMS agencies [8–11].

Despite this, uncertainty remains over the favourability of ketamine’s haemodynamic profile versus alternative induction agents; the literature contains contradictory results against drugs such as etomidate [5, 12–14], fentanyl [15] and midazolam [16, 17]. Given the high stakes of RSI procedures, determining the optimal agent to induce emergency anaesthesia is a matter of priority for the emergency medical community.

## Aims

The aim of this systematic review and meta-analysis is to evaluate the effect of ketamine on incidence of PIH following emergency RSI when compared with alternative induction agents, thus contributing to the body of evidence determining the optimal induction agent for this purpose.

## Methods

This systematic review and meta-analysis was registered with the International Prospective Register of Systematic Reviews (PROSPERO – CRD42024494085). It has been reported in line with PRISMA guidelines [18], demonstrated in Appendix 1. Ethical approval was not required.

### Data sources

Searches were conducted on PubMed, Embase, Scopus, the Cochrane Library, and ClinicalTrials.gov, closing on 26th February 2024. Search strategies combined 3 key concepts: RSI, ketamine, and hypotension, making use of medical subject headings where available. Exact search terms varied according to database; full strategy is available in Appendix 3.

### Eligibility

Studies eligible for this review fulfilled criteria for their population, intervention, comparator, and outcome.

i) *Population*: Eligible studies included adult patients undergoing emergency RSI, defined as RSI for any purpose besides facilitating non-emergency surgery. Requirement for RSI under these circumstances was considered indicative of high clinical acuity, including medical, surgical and trauma patients as well pre-hospital and in-hospital settings. Pre-existing haemodynamic instability (including vasopressor requirement) was not necessary for inclusion, but where present was considered a proxy for the emergent nature of intubation if eligibility was otherwise unclear. Studies exclusively studying children were excluded, although for all others a lower limit for minimum participant age was not enforced.
ii) *Intervention*: Eligible studies contained a study arm specifying administration of any dose of ketamine equal to or greater than 0.5 mg/kg for the purpose of inducing anaesthesia to facilitate endotracheal intubation. Co-administration of other agents was acceptable, excluding studies investigating “ketofol” (pre-mixed ketamine and propofol). Both ketamine dosage and co-induction agents could be pre-specified or selected at the administrator’s discretion without affecting eligibility for this review.
iii) *Comparator*: Eligible studies included patients receiving at least one alternative induction agent to ketamine.
iv) *Outcome*: Eligible studies measured dichotomous incidence of post-induction hypotension. No single definition was required for inclusion. Acceptable definitions included absolute or relative change in blood pressure (either systolic blood pressure (SBP) or mean arterial pressure (MAP)) versus baseline, requirement for resuscitation (including fluid boluses, or initiation or escalation of vasopressors), or combinations of these. No single time interval in which the PIH definition was to be met was required for this review.

### Study selection

All search results were compiled into an Excel (Microsoft) spreadsheet, which was independently reviewed by each author. Disagreements regarding eligibility were to be arbitrated by an independent third party.

All results were screened at abstract level; duplicates and those failing to fulfil population, intervention, comparator or outcome criteria were excluded at this stage. The remainder were reviewed in full-text, reviewing Supplements where possible and relevant, to confirm fulfilment of eligibility criteria and determine presence of extractable data.

### Risk of bias

Risk of bias tools were applied to all included studies: Cochrane’s RoB-2 tool to randomised controlled trials (RCTs) [19] and ROBINS-I to observational studies [20]. Critical appraisal of each paper manually highlighted studies at greater risk of bias.

### Data extraction

Frequency of PIH in ketamine and non-ketamine groups was extracted from each study. Where this information was not directly presented, it was calculated from data available in the full text or available Supplements. Where it was possible to isolate data from patients receiving no induction agent (drug-free or paralytic-only intubation), these were excluded from the non-ketamine group. Where multiple outcome measures indicative of post-induction hypotension were presented, one was selected subjectively on grounds of clinical relevance for imputation into the meta-analysis. Data were compiled in an Excel spreadsheet, with odds ratios (ORs) and standard errors of their natural logarithms manually calculated.

Where studies presented adjusted odds ratios generated by statistical techniques such as logistic regression or propensity matching, these were preferentially imputed into meta-analysis over values calculated from raw frequency data. In such cases, standard errors were calculated from reported confidence intervals (CIs), taking means of those derived from their upper and lower bounds to minimise rounding error.

Where raw data was insufficient to isolate effects of ketamine or compute values necessary for inclusion in meta-analysis (such as standard error), Supplements were searched by hand.

### Statistical analysis

SPSS version 29.0.1.0 (IBM) was used to carry out statistical analysis and generate statistical diagrams.

i) Meta-analysis

A random-effects model was used to generate a pooled estimate of OR of PIH with ketamine versus comparators, alongside 95% confidence interval and 95% prediction interval. The Knapp-Hartung adjustment of standard errors was applied. Pre-determined sensitivity analyses were conducted:

- pooled effect size estimate using only unadjusted data;
- pooled effect size estimate from randomised controlled trials (RCTs);
- pooled effect size estimate from RCTs not at high risk of bias;
- pooled effect size estimate from all studies not at high risk of bias.

A trim-and-fill analysis was carried out to illustrate the effect of publication bias in the literature on meta-analysis output.

ii) *Subgroup analysis*

Four subgroup analyses were carried out:

- by comparator induction agent;
- with vs without pre-existing haemodynamic instability;
- medical patients vs trauma patients;
- pre-hospital vs in-hospital RSI.

For each analysis, every study was designated as either belonging to one subgroup, or as containing mixed data. In the latter case, data for each subgroup were extracted individually; in all such cases, an unadjusted OR was imputed into the meta-analysis regardless of statistical technique used by the parent study. Where extraction from “mixed” studies was not possible for lack of relevant raw data, the study was excluded from the corresponding subgroup analysis.

## Results

### Study selection

**Fig. 1:**
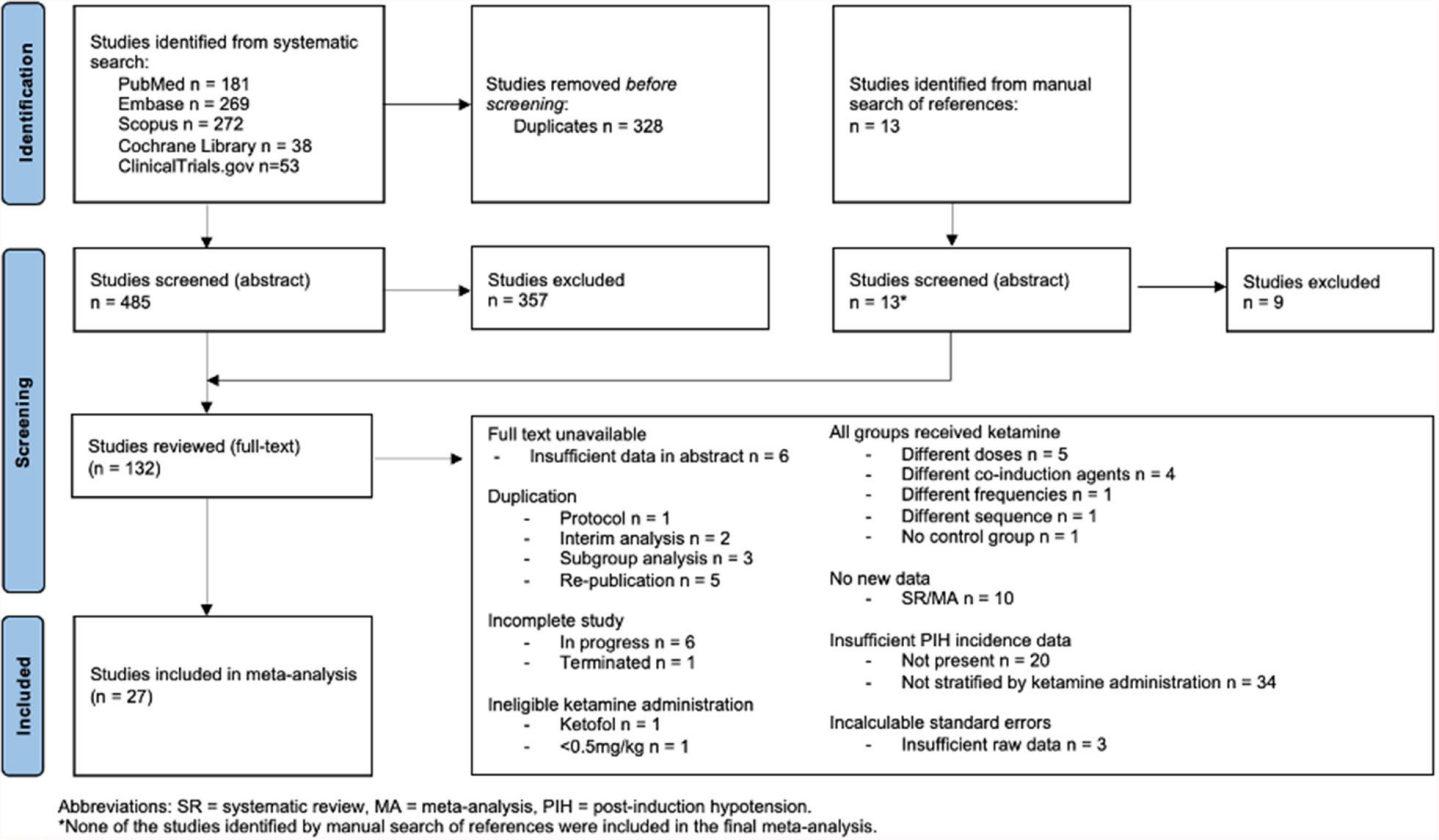
Flow diagram demonstrating selection of studies into the meta-analysis

A total of 826 search results were considered, including 813 from systematic search. Of these, 132 were reviewed in full-text, with 27 studies (all originating from systematic search) eligible for inclusion into the final analysis. Figure 1 illustrates the sources of these results and reasons for rejection. There were no cases of disagreement around final eligibility between the two reviewers.

The 27 final studies included 31,956 patients analysed for PIH incidence, of which 8,472 received ketamine and 23,484 were classed as controls. Their characteristics are presented in Table 1. Further critical appraisal relevant to the aims of this review is presented in Appendix 5.

**Table 1:** Characteristics of included studies

Study	Design	Population		Ketamine		Comparator			PIH Definition^‡^
		Patients	Setting	Dose	Baseline	Agent	Dose	Baseline	
Ali et al. [15]	RCT	Sepsis (on NA infusion)	OR	1 mg/kg	MAP 74 SI 1.1	Fentanyl	2.5 μg/kg	MAP 67 SI 1.2	δMAP > 20% (10 min)
Zuin et al. [16]	OCS	STEMI (SBP > 90)	CCL	1 mg/kg	SBP 102 SI 1.02	Midazolam	0.3 mg/kg	SBP 103 SI 1.02	SBP < 90 or δ > 20% (10 min)
Kang et al. [29]	OCS	Critically Ill	ICU	–	–	Etomidate	–	–	–
Van Berkel et al. [26]	OCS	Sepsis (survived > 24h)	ICU	1.8 mg/kg*	SBP 108 MAP 78	Etomidate	0.3 mg/kg*	SBP 120 MAP 85	(MAP < 70 + δ > 40%), MAP < 60, (new pressor or dose δ > 30%) (24h)
Kunkel and Lenz [54]	OCS	Requiring RSI by HEMS	PH	100 mg*	SBP 112	Etomidate	30 mg*	SBP 135	SBP < 90, δ > 5mmHg if baseline < 90 (10 min)
Ishimaru et al. [28]	OCS	Emergency (SI > 0.9)	ED	–	–	Midazolam Propofol	–	–	SBP < 90 (30 min) or δ > 20% (immediately)
Breindahl et al. [22]	OCS	Trauma	PH or ED within 30 min	75 mg*	SBP 121 ISS 27	Propofol	100 mg*	SBP 130 ISS 25	Vasopressor use
Grant et al. [41]	OCS	Emergency (SBP > 90)	ED	–	–	Fentanyl Midazolam Propofol	–	–	SBP < 90 or δ > 20mmHg (1h)
Tangkulpanich et al. [31]	OCS	Sepsis (SIRS)	ED	–	–	Fentanyl, Midazolam, Etomidate, Propofol, Diazepam	–	–	SBP < 90, δSBP > 20%, MAP < 65, vasopressor use, > 30ml/kg fluid (1 h)
Hsieh et al. [27]	OCS	Emergency (SBP < 90 or MAP < 65)	ED	–	–	Etomidate	–	–	SBP < 90 or MAP < 65
Srivilaithon et al. [33]	RCT	Sepsis	ED	1–2 mg/kg	SBP 118	Etomidate	0.2–0.3 mg/kg	SBP 113	SBP < 90 or MAP < 65
Nakornchai et al. [30]	OCS	Emergency (non-trauma)	ED	–	–	Midazolam, Etomidate, Propofol, Diazepam	–	–	SBP < 90 or δ > 20% (10 min)
Bakhsh et al. [43]	OCS	Emergency (vasopressors not required)	ED	90 mg*	SBP 149 MAP 102	Etomidate	22.6 mg*	SBP 138 MAP 102	δSBP > 20% (10 min)
Knack et al. [34]	RCT	Emergency	ED	2 mg/kg*	SBP 139	Etomidate	0.27 mg/kg*	SBP 140	SBP < 90 within ED stay
Stanke et al. [13]	OCS	Requiring RSI by HEMS	PH	1.9 mg/kg*	SBP 132 MAP 99	Etomidate	0.3 mg/kg*	SBP 140 MAP 101	δSBP > 20% (15 min)
King et al. [17]	OCS	ROSC (following medical CA)	PH	1 mg/kg*	SBP 130 SI 0.80	Midazolam	0.03 mg/kg*	SBP 132 SI 0.84	SBP < 90, δ > 10% if baseline < 90 (30 min)
Mattson et al. [44]	OCS	Emergency	ED	–	–	Etomidate Propofol	–	–	SBP < 100 (30 min)
Matchett et al. [12]	RCT	Critically Ill	ICU	1.1 mg/kg*	SBP 120 MAP 90	Etomidate	0.2 mg/kg*	SBP 121 MAP 89	SBP < 65, new pressor or ↑dose, CA (1h)
Foster et al. [51]	OCS	Emergency	ED	–	SBP 121 MAP 90 SI 0.97	Etomidate	–	SBP 134 MAP 98 SI 0.83	SBP < 90 or MAP < 65 (20 min)
Kim et al. [42]	OCS	Emergency	ED	1.2 mg/kg*	–	Etomidate Others (not specified)	0.29 mg/kg* –	–	SBP < 90, MAP < 65, δSBP/MAP > 20% if baseline < 90/65, new pressor
Driver et al. [46]	OCS	Emergency	ED	1.33 mg/kg*	–	Etomidate	0.28 mg/kg*	–	SBP < 100 (15 min)
Kuza et al. [23]	OCS	Trauma (within 24h admission)	ED/OR	–	SBP 121 SI 0.81 ISS 22	Etomidate Propofol	–	SBP 135 SI 0.72 ISS 16	Vasopressor use (15 min)
Price et al. [24]	OCS	Requiring RSI by HEMS	PH	1.2 mg/kg*	SBP 139	Etomidate	0.3 mg/kg*	SBP 134	SBP < 90 or MAP < 60 (within 5 readings, median 28 min)
Pollack et al. [25]	OCS	Requiring RSI by HEMS	PH	–	–	Fentanyl Midazolam Etomidate	–	–	–
Nakajima et al. [21]	RCT	Emergency	ED	2 mg/kg	SBP 136	Etomidate	0.3 mg/kg	SBP 128	δSBP > 20% (15 min)
Lyon et al. [11]	OCS	Trauma	PH	2 mg/kg or 1 mg/kg	SBP 133 MAP 102 ISS 26	Etomidate	0.3 mg/kg or 0.15 mg/kg	SBP 129 MAP 98 ISS 22	SBP < 90 or δSBP > 20% or δMAP > 20% (5 min)
Elsherbiny et al. [32]	RCT	Septic shock (Sepsis-3 definition)	OR	1 mg/kg	MAP 78	Thiopental	2 mg/kg	MAP 77	δMAP > 20% and (↑dose pressor or ↓dose volatile anaesthetic) (14 min)

^*^Mean, median or mode dose administered; the remaining studies specified dosages to be given to all patients; ‡With time point if available; All Baseline data are means or medians; “–" data not available

*PIH* post-induction hypotension, *RCT* Randomised Controlled Trial, *NA* noradrenaline, *OR* operating room, *MAP* mean arterial pressure, *SI* shock index, *OCS* Observational Cohort Study, *STEMI* ST-elevation myocardial infarction, *CCL* cardiac catheterisation laboratory, *SBP* systolic blood pressure, *ICU* intensive care unit, *RSI* rapid sequence induction, *HEMS* helicopter emergency medical service, *PH* pre-hospital, *ED* emergency department, *ISS* injury severity score, *SIRS* systemic inflammatory response syndrome, *ROSC* return of spontaneous circulation, *CA* cardiac arrest

No additional extractable data was found within the Supplements of papers reviewed in full-text.

### Risk of Bias

i) *Randomised Controlled Trials*: Risk of bias for the 6 RCTs was assessed using Cochrane’s RoB-2 tool, which did not identify any studies at low overall risk of bias. One (Nakajima et al. [21]) was identified as at high risk, largely on account of its alternate-day randomisation process. This study was thus excluded from the relevant sensitivity analyses. Full breakdown of each domain is presented in Table 2.
ii) *Observational Studies*: Cochrane’s ROBINS-I tool was used to identify areas in which the 21 included observational cohort studies (OCSs) were at risk of bias. Breakdown of each of its domains is shown in Table 3. Not unexpectedly in observational studies of emergency patients, no study was found at low risk of bias. All studies were at high risk in at least one domain; thus, all studies were designated as at high overall risk. The tool’s discriminatory power was therefore poor for this set of studies; the planned sensitivity analysis instead excluded 5 papers with manually identified methodological flaws: Breindahl et al. [22], Kuza et al. [23], Price et al. [24], Pollack et al. [25], and Van Berkel et al. [26]. The relevant critical appraisal leading to their selection is presented in Appendix 5.

**Table 2:**
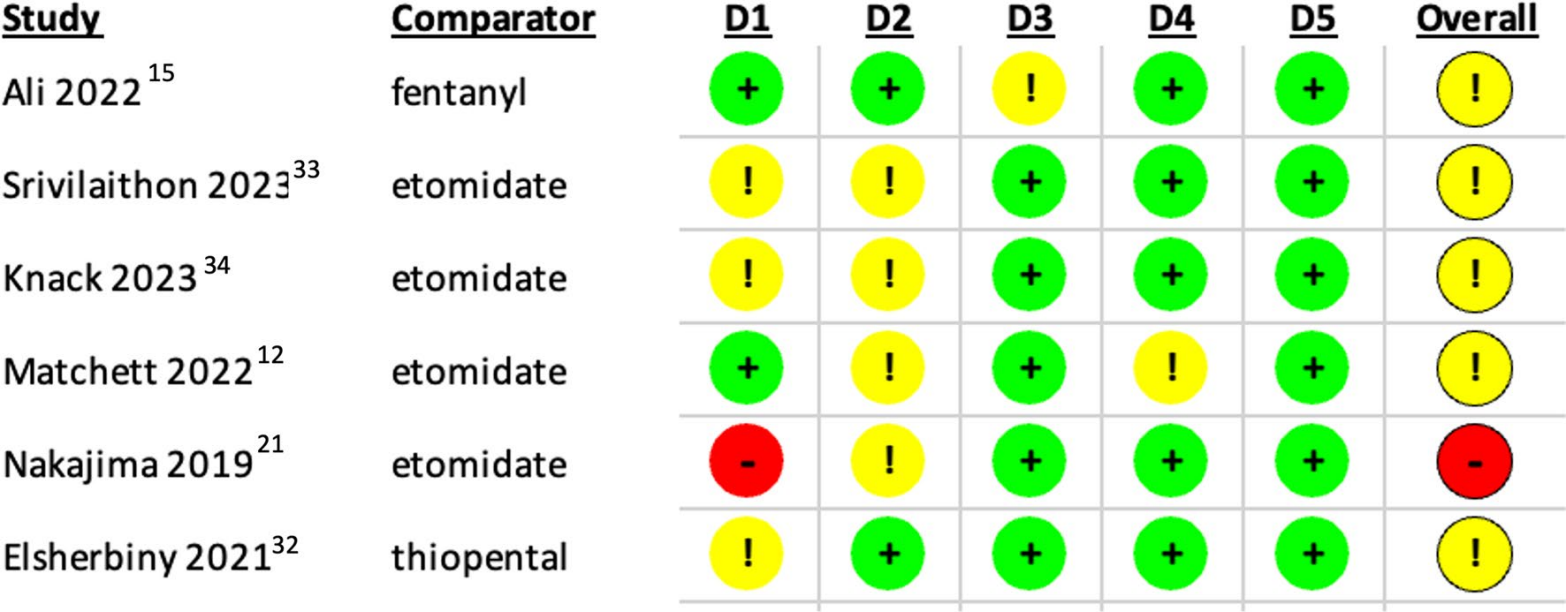
RoB-2 domains and overall risk of bias for the 6 RCTs included in this review

D1 (Domain 1): Randomisation process. D2 (Domain 2): Deviations from intended interventions. D3 (Domain 3): Missing outcome data. D4 (Domain 4): Measurement of the outcome. D5 (Domain 5): Selection of the reported result

**Table 3:**
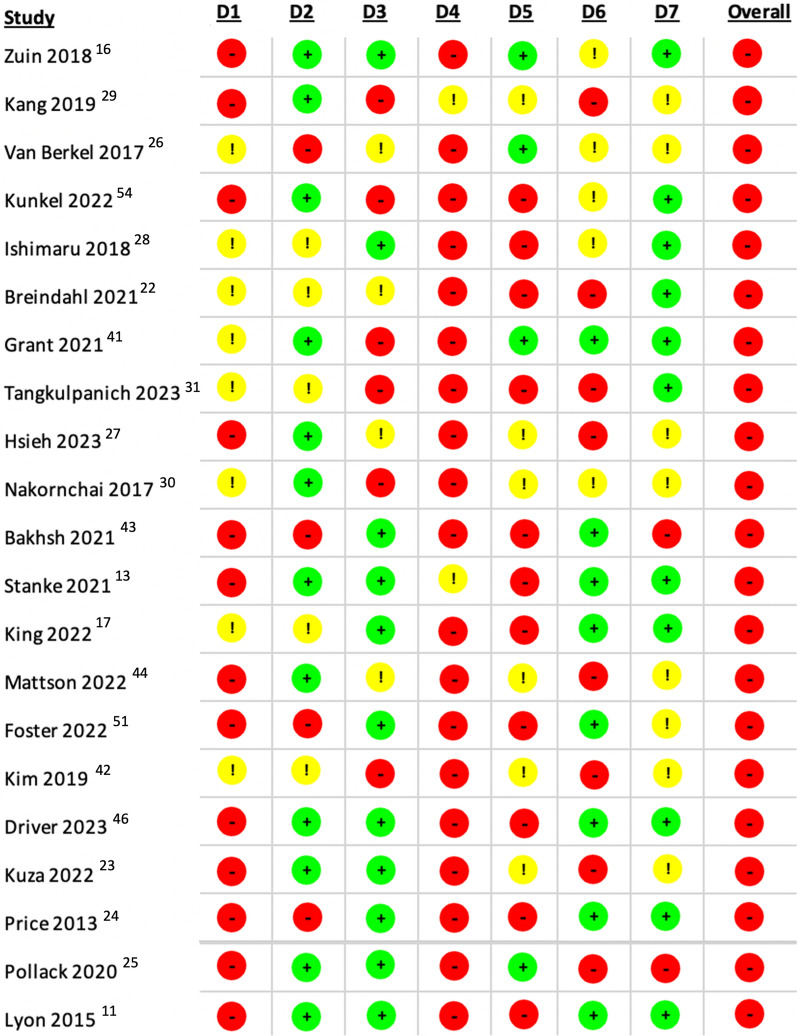
ROBINS-I domains and overall risk of bias for the 21 OCSs included in this review

D1 (Domain 1): Bias due to confounding. D2 (Domain 2): Bias in selection of participants into the study. D3 (Domain 3): Bias in classification of interventions. D4 (Domain 4): Bias due to deviations from intended interventions. D5 (Domain 5): Bias due to missing data. D6 (Domain 6): Bias in measurement of outcomes. D7 (Domain 7): Bias in selection of the reported result

### Primary analysis

The random-effects model with the Knapp-Hartung adjustment of standard error produced a pooled estimate of odds ratio of PIH following emergency RSI with ketamine versus other induction agents of 1.10, as demonstrated in Fig. 3. The 95% confidence interval is 0.78–1.56, with p-value 0.58. There was evidence of significant heterogeneity in results, with p-value < 0.001 for Q, and I^2^ 0.90. The 95% prediction interval is 0.24–4.98.

### Sensitivity analyses

4 sensitivity analyses were carried out:

i) Meta-analysis imputing only the raw frequency data from each study, with no adjustment of OR. Adjusted data from 4 studies (Hsieh et al. [27], Ishimaru et al. [28], Kang et al. [29], Van Berkel et al. [26]) had been imputed into the primary analysis; replacing these with unadjusted figures resulted in a pooled OR of 1.14 (0.82–1.57).
ii) Meta-analysis exclusively including the 6 RCTs produced a pooled OR of 1.43 (0.37–5.49).
iii) Meta-analysis exclusively including the 5 RCTs not at high risk of bias produced a pooled OR of 1.14 (0.26–4.95).
iv) Meta-analysis excluding all studies (1 RCT and 5 OCSs) at greater risk of bias produced a pooled OR of 0.99 (0.69–1.43).

### Publication bias

**Fig. 2:**
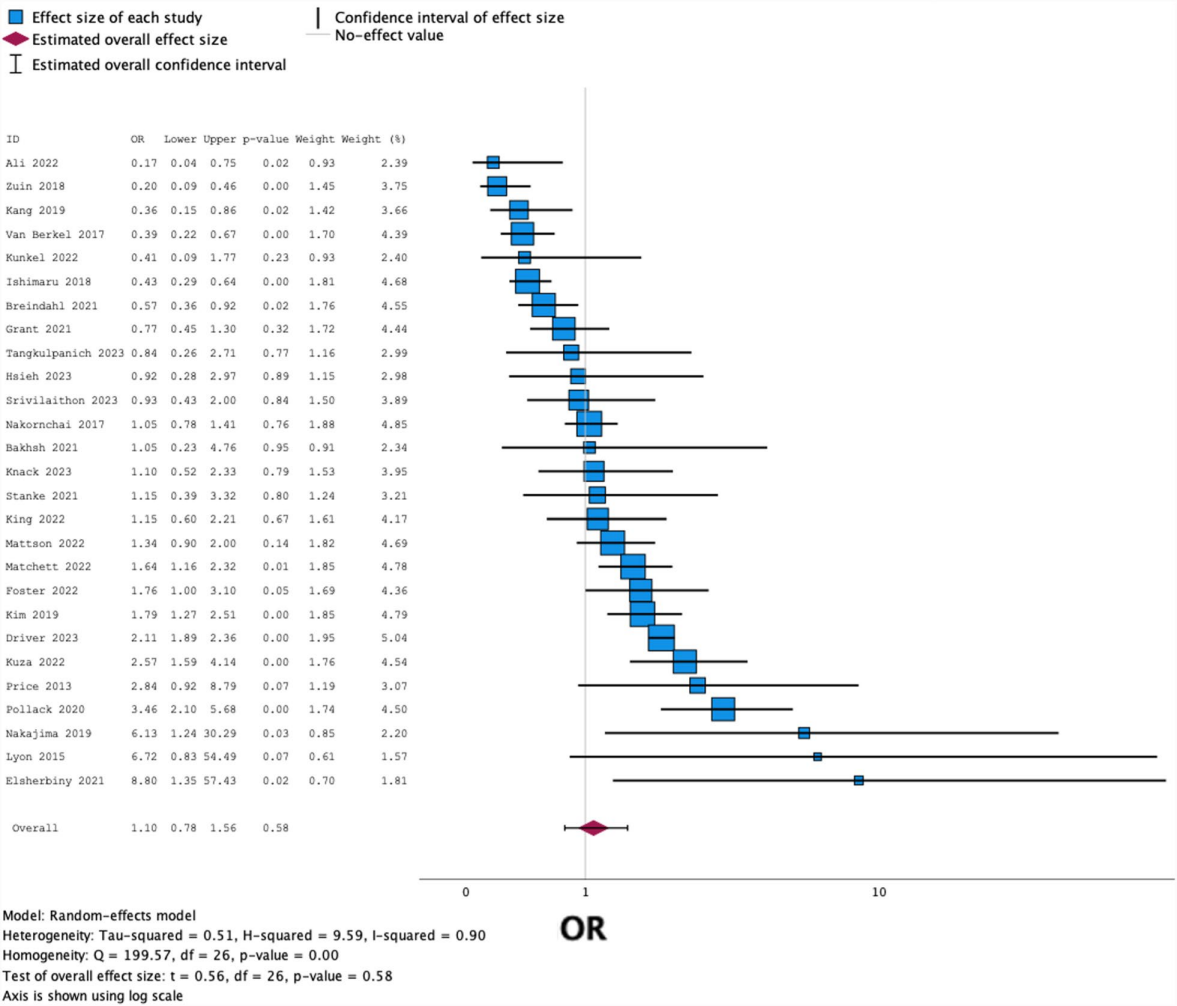
Forest plot showing the OR for PIH (with 95% CI) in all 27 included studies, with a pooled estimate

**Fig. 3:**
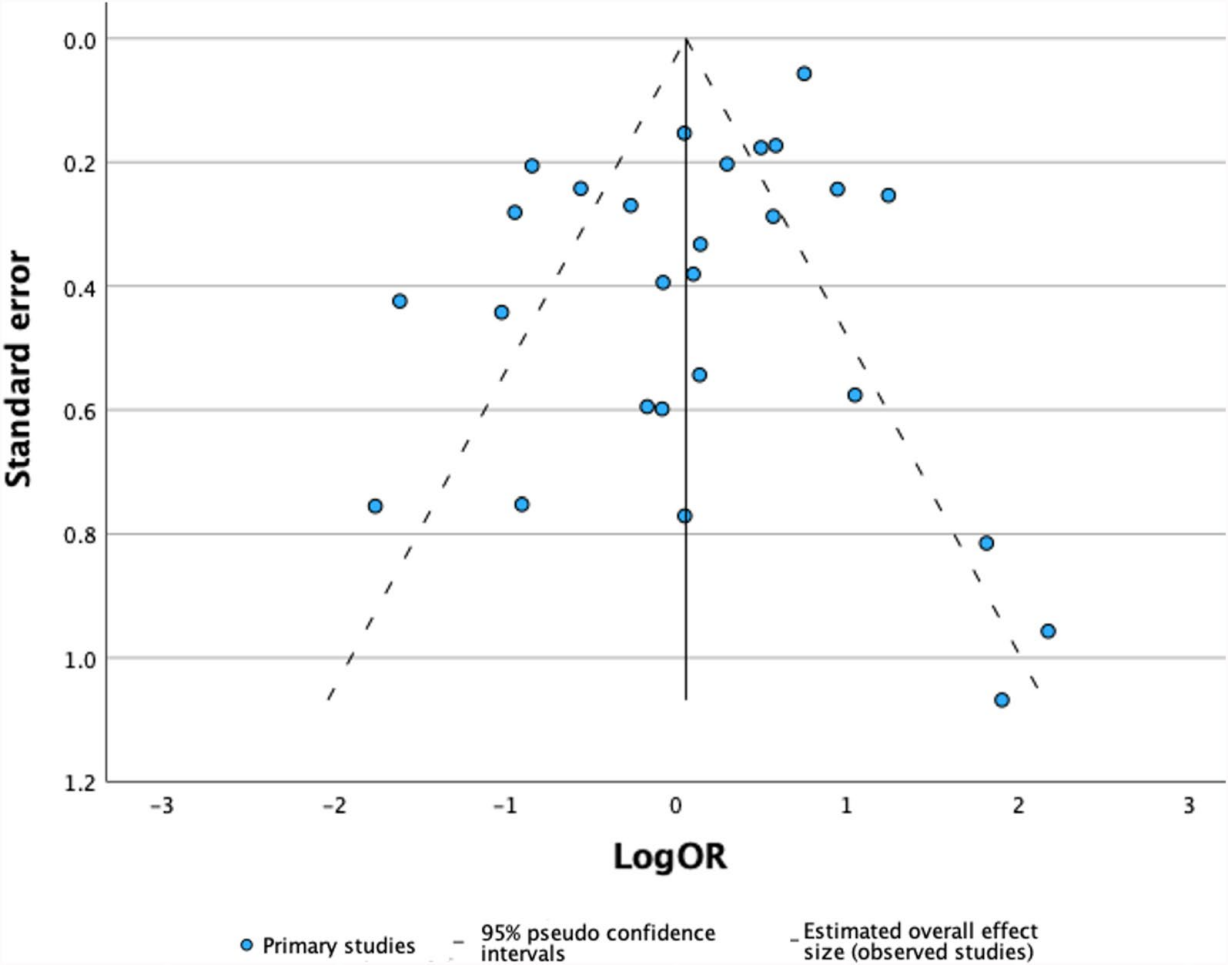
Funnel plot with all 27 included studies

A Funnel plot of the 27 included studies is shown in Fig. 2. Trim-and-fill analysis identified 1 missing study, altering the OR to 1.06 (0.74–1.51) when imputed alongside the 27 observed studies. Furthermore, Egger’s regression-based test produced an intercept of -0.008 with p-value 0.982 (Fig. 3).

### Subgroup analyses

4 subgroup analyses were carried out. Data extraction is described in Appendix 6.

**Fig. 4:**
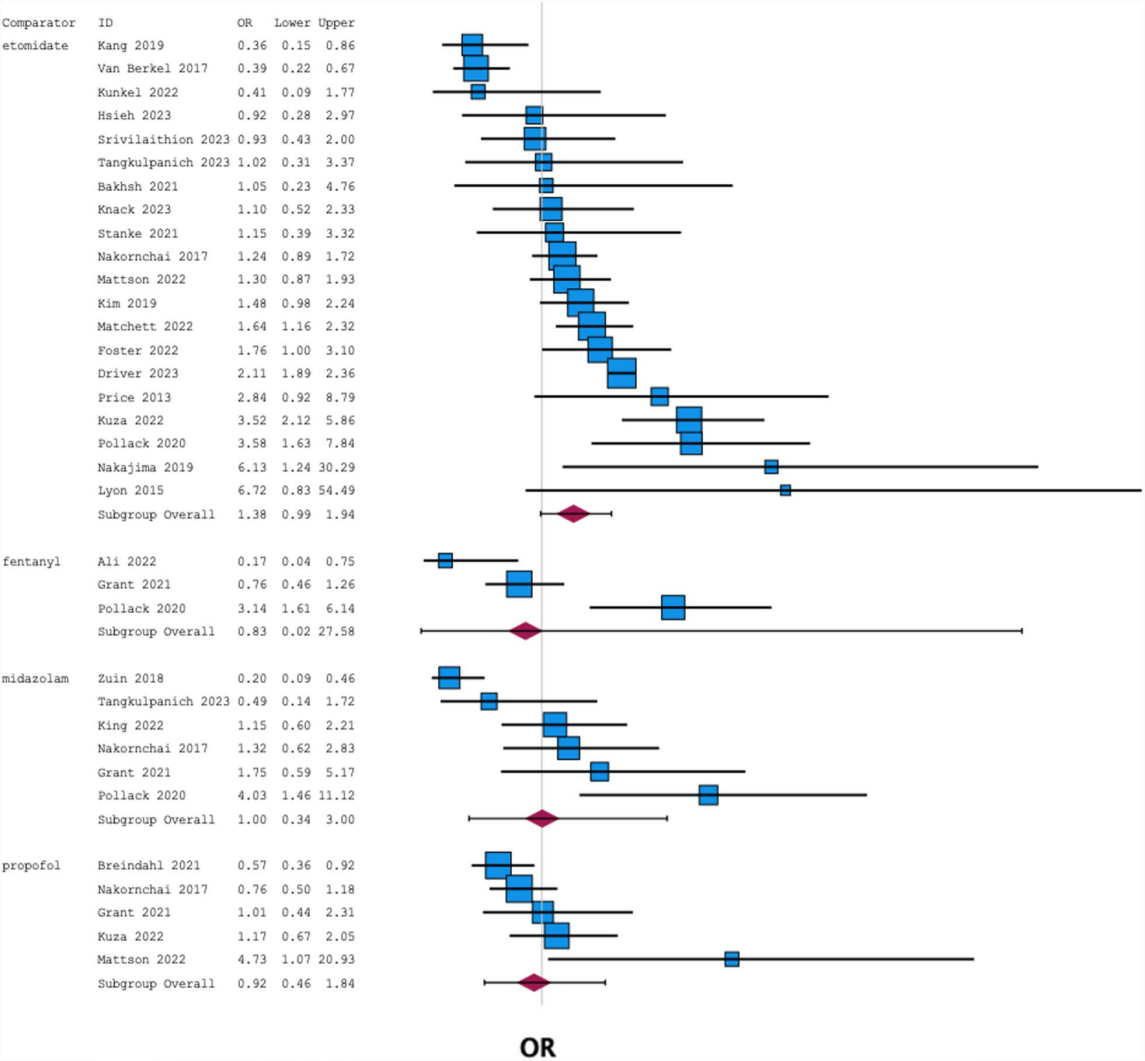
Forest plot showing the pooled OR for PIH (with 95% CI) for ketamine against each comparator agent

i) Meta-analysis comparing ketamine to individual comparator induction agents. 6 total comparators were used in eligible studies: diazepam, etomidate, fentanyl, midazolam, propofol, and thiopental. Diazepam and thiopental were investigated in only 2 and 1 studies respectively [30–32] and were thus excluded. Data for the remaining agents are demonstrated in Fig. 4 and Table 4.
ii) Effect on PIH incidence of ketamine versus other induction agents was assessed separately in patients with and without pre-existing haemodynamic instability. OR with evidence of haemodynamic instability was 1.10 (0.36–3.36) versus 1.02 (0.46–2.27) without.
iii) In medical patients, OR of PIH when inducing with ketamine was 0.88 (0.42–1.87) versus 1.25 (0.50–3.09) in trauma patients.
iv) In RSIs conducted pre-hospital, OR of PIH when inducing with ketamine was 1.76 (0.72–4.33) versus 0.99 (0.67–1.47) for RSIs conducted in hospital.

**Table 4:** OR for PIH when ketamine is used for emergency RSI versus specific comparators, with 95% CIs and 2-tailed p-values

Comparator	Odds ratio	Lower 95% CI	Upper 95% CI	*p*-value
Etomidate	1.38	0.99	1.94	0.058
Fentanyl	0.83	0.02	27.58	0.837
Midazolam	1.00	0.34	3.00	0.992
Propofol	0.92	0.46	1.84	0.181

*OR *odds ratio, *CI* confidence interval

### Summary

Meta-analysis demonstrated no significant difference in incidence of PIH following emergency RSI with ketamine as the induction agent versus other drugs. Sensitivity analyses all supported this result. No subgroup reached statistical significance in the difference in PIH incidence.

## Limitations

### Characteristics of included studies

Only 6 of 27 included studies were RCTs [12, 15, 21, 32–34]; 95.9% of the 31,956 data points were derived from observational research. Methodological flaws are to be expected when investigating unpredictable & complex emergency situations; indeed, 22 of the 27 studies were found to be at high risk of bias.

Selection of studies for inclusion in the review was vulnerable to sampling bias. Some of those reviewed in full-text reported incidence of adverse events, including PIH, with insufficient raw data (including within Supplements) from which to divide these by induction agent [35–37]. Others reported continuous variables such as absolute or percentage change in SBP or MAP, without dichotomous incidence data [14, 38]; examples are presented in Appendix 4. The possibility remains that exclusion of these studies systematically affected the final sample and thus the result.

### Methodological heterogeneity between included studies

The sample of studies selected for this review reflect the considerable methodological heterogeneity in the wider literature. Inclusion criteria, study arm protocols and outcome measures vary widely; few studies answer precisely the same question. This review’s broad eligibility criteria achieve a large sample size, aiming to capture the effect of ketamine on PIH incidence in a manner indicative of real-world use, accepting the consequent risk of introducing bias by systematically or disproportionately emphasising results from certain clinical scenarios. It is likely that, for some circumstances, external validity suffers.

A particular source of heterogeneity between included studies is their comparator drugs. This review compared ketamine against any alternative; no such work has previously been undertaken, and by including subgroup analysis, this is potentially hypothesis-generating. Although wide variation exists among induction regimes used for the control arm of this analysis, no agent was found to produce a statistically significant difference in OR of PIH compared to ketamine in the subgroup analysis.

This review includes medical and trauma patients, within both of which exist further unstudied subgroups including presence and aetiology of haemodynamic shock. Furthermore, these groups are likely to differ in terms of co-morbidities and physiological reserve [39, 40]. The review also includes patients with and without pre-existing haemodynamic instability, who may not only respond differently to induction drugs but experience distinct clinical consequences as a result.

Not all included studies reported ketamine dosage, with considerable variation present between and within those that did, ranging from as little as 0.5 mg/kg to in excess of 2 mg/kg [41, 42]. Additionally, regimes varied between fixed and discretionary doses [11, 15, 43]. As increased dosage of ketamine is postulated to independently increase risk of post-induction haemodynamic changes [44–46], this heterogeneity might have impacted meta-analysis results.

No single definition of PIH was replicated across more than 2 studies out of the final 27. As there is no standardised or universally agreed-upon definition [47], it fell to each individual group of researchers to devise a definition suiting their population and aims. Furthermore, the time interval within which hypotension was considered associated with or attributable to induction agents varied significantly. This review is therefore limited by the current lack of PIH definition supported by any level of evidence beyond expert opinion.

### Confounding

Degree to which patients were resuscitated prior to RSI was often unclear. Administration of fluid or vasoactive drugs, as well as measures such as pre-oxygenation, whether carried out or omitted despite being indicated, are likely to affect both baseline and post-induction haemodynamics. This potentially confounds the meta-analysis results.

During RSI, pre-treatment or co-induction agents such as fentanyl or midazolam were frequently administered alongside ketamine; some [17, 24], but not all [26], studies reported comparable frequencies and doses between study arms. As fentanyl has been demonstrated to exert independent haemodynamic effects [48], those of ketamine or a comparator can be difficult to isolate. Analysing fentanyl and ketamine separately does not apply to all relevant real-world clinical situations.

Post-induction, patient factors necessitating variations in treatment (such as positive pressure ventilation strategies [49, 50]) can also confound haemodynamic outcome measures. This is particularly true for studies including vasopressor administration within their PIH definition, as threshold for such intervention is unlikely to be consistent within or between studies.

### Methodological flaws in included studies

The meta-analysis was also limited by the issue of confounding by indication. Many eligible observational studies found ketamine more likely to be administered in patients with signs of haemodynamic instability [23, 26, 51]. It is therefore possible that the analysis was biased towards an increased PIH incidence with ketamine, in fact reflecting a more unstable cohort. However, sensitivity analyses isolating results from RCTs mirrored the overall result, suggesting this had little impact.

Subgroup analysis aimed to answer the study question separately within pre-specified groups, however was hampered by some studies not presenting sufficient raw data from which to separate PIH incidence between subgroups (detailed in Appendix 6). It remains possible that the data discarded might have been so systematically, introducing bias.

Many studies had a degree of crossover between groups. Some presented PIH data dichotomously divided into patients receiving versus not receiving each drug, without accounting for those administered more than one agent [30, 41]. Others considered fentanyl, in particular, a pre-treatment drug or analgesic [25, 43], rather than an induction agent, introducing further bias affecting PIH outcomes.

## Discussion

### Heterogeneity in results

Methodological heterogeneity between eligible studies is reflected in heterogeneity of their results (I^2^ = 90%). This review’s broad eligibility criteria produced a pooled estimate of OR of PIH not necessarily applicable to all situations in which RSI is indicated. However, subgroup analyses did not identify any specific areas in which this is the case; only comparison of ketamine to etomidate approached significance.

Ketamine was found to be a safe alternative to etomidate in settings as varied as pre-hospital trauma patients [13] versus septic medical intensive care patients [26, 29]. Studies reporting significantly increased PIH incidence following RSI with ketamine are equally diverse, including the largest RCT in this review [12], which defined PIH with the less frequently used but more clinically consequential Vanderbilt definition of cardiovascular collapse [52, 53] in a mixed critically ill population.

These results suggest that clinical scenarios in which choice of primary induction agent affects PIH incidence are nuanced and as yet incompletely described. Confounding factors affecting a situation as complex as emergency RSI will need further elucidation prior to issuing firm recommendations on agent choice in any given circumstance.

### Clinical relevance of PIH

Lack of unified definition of PIH reflects uncertainty regarding what constitutes clinically relevant hypotension. Some studies applied distinct definitions of PIH depending on pre-induction haemodynamics [17, 42, 54], illustrating that a given absolute reduction in blood pressure can have greater relevance in hypotensive or shocked patients.

Furthermore, acute reduction in blood pressure is not necessarily harmful if indicative of blunting of the harmful hypertensive response to laryngoscopy [11]. In particular, the analgesic effect of fentanyl has been postulated to exert benefit through this effect [48]. This is commonly co-administered alongside ketamine, for example in “3:2:1” regimens advocated by UK HEMS agencies for pre-hospital trauma RSI.

These nuances of peri-intubation blood pressure will require consideration if a standardised definition of PIH is to be agreed. Given the frequent co-administration of multiple agents to achieve the goals of RSI, further research should ideally focus on regimens rather than individual agents, in order to increase external validity to real-world practice.

### Mechanisms of PIH caused by ketamine

Ketamine has been proposed to contribute to PIH by various mechanisms. For instance, its direct myocardial depression might outweigh sympathomimetic-driven increases in systemic vascular resistance in critically ill patients with depleted catecholamine reserves [5, 6, 55, 56]; this is not supported by this review’s finding that the effect of ketamine on PIH incidence was similar in subgroups with or without pre-existing haemodynamic instability.

Alternatively, its sympathomimetic action might paradoxically inhibit cardiac function through increased myocardial oxygen demand [7] and thus ischaemia, particularly in elderly or more co-morbid populations. Although this review did not specifically consider co-morbidities or pre-existing cardiovascular disease, this mechanism is not supported by the finding that the effect of ketamine on PIH incidence was not greater in medical patients versus the trauma population.

### Link to patient-centred outcomes

It was beyond the scope of this review to consider patient-centred outcomes. Lack of universal definition notwithstanding, PIH has been proven an independent risk factor for mortality and prolonged admission [2, 3]. The extent to which these outcomes can be improved with the optimisation of induction drug regimens remains unclear.

## Conclusion

In this methodologically heterogeneous sample of studies, preferential use of ketamine during rapid sequence induction did not significantly alter incidence of post-induction hypotension. Further research is required to determine the optimal choice of induction agent in a variety of distinct clinical situations in order to minimise PIH incidence and improve patient outcomes. Ideally, this should be based on a consensus definition of PIH, which does not currently exist.

Given the extent of this heterogeneity in the published literature, it is not currently possible to draw further specific conclusions.

## Supplementary Information

Supplementary material 1

## Data Availability

The full list of search results is available on request from the corresponding author.

## Abbreviations

CA: Cardiac arrest
CCL: Cardiac catheterisation laboratory
CI: Confidence interval
ED: Emergency department
HEMS: Helicopter emergency medical service
ICU: Intensive care unit
ISS: Injury severity score
MA: Meta-analysis
MAP: Mean arterial pressure
NA: Noradrenaline
NMDA: N-methyl-D-aspartate
OCS: Observational cohort study
OR: Odds ratio
OR: Operating room
PH: Pre-hospital
PIH: Post-induction hypotension
PRISMA: Preferred reporting items for systematic reviews and meta-analyses
PROSPERO: International prospective register of systematic reviews
RCT: Randomised controlled trial
RoB-2: Risk of bias 2
ROBINS-I: Risk of bias in non-randomised studies of interventions
ROSC: Return of spontaneous circulation
RSI: Rapid sequence induction
SBP: Systolic blood pressure
SI: Shock index
SIRS: Systemic inflammatory response syndrome
SPSS: Statistical product and service solutions
SR: Systematic review
STEMI: ST-elevation myocardial infarction
