# Supplementary material for: Incidence of post-induction hypotension following emergency rapid sequence induction with ketamine: a systematic review and meta-analysis
Source: Scand J Trauma Resusc Emerg Med. 2025 May 1;33:71. doi: 10.1186/s13049-025-01374-7 (PMC12044812; doi:10.1186/s13049-025-01374-7)
Supplement: Supplementary file 1 — Supplementary material 1 [file 13049_2025_1374_MOESM1_ESM.docx]

**Appendices** to “Incidence of Post-Induction Hypotension following Emergency Rapid Sequence Induction with Ketamine: a Systematic Review and Meta-Analysis”

Dr Pedro Vila de Mucha^1^ 0009-0005-2378-2724

Professor Stephen Thomas^2^ 0000-0003-2833-5921

^1,2^ Blizard Institute, Queen Mary University of London, London, UK

*Correspondence to Dr Vila at:*

pedro.vilademucha@nhs.net

**Table of Contents**

| List of Abbreviations |  | 3 |
| --- | --- | --- |
|  |  |  |
|  |  |  |
| Appendix 1 | *PRISMA Checklist* | 4 |
| Appendix 2 | *Supplementary Background* | 8 |
| Appendix 3 | *Search Strategies* | 9 |
| Appendix 4 | *Examples of Rejected Studies* | 13 |
| Appendix 5 | *Critical Appraisal of Eligible Studies* | 14 |
| Appendix 6 | *Data Extraction for Subgroup Analyses* | 23 |

*NB References within these Appendices are listed on p29 of the primary manuscript.*

**List of Abbreviations**

| ACS | Acute Coronary Syndrome |
| --- | --- |
| BP | Blood Pressure |
| ED | Emergency Department |
| HR | Heart Rate |
| MAP | Mean Arterial Pressure |
| NEAR | National Emergency Airway Registry |
| OCS | Observational Cohort Study |
| OR | Odds Ratio |
| PIH | Post-Induction Hypotension |
| PRISMA | Preferred Reporting Items for Systematic Reviews and Meta-Analyses |
| RCT | Randomised Controlled Trial |
| ROSC | Return of Spontaneous Circulation |
| RSI | Rapid Sequence Induction |
| SBP | Systolic Blood Pressure |
| SI | Shock Index |
| SPP | Scientific Paper & Presentation (Paper) |
| STEMI | ST-Elevation Myocardial Infarction |

**Appendix 1: PRISMA Checklist**

Table 1 demonstrates a completed checklist according to Preferred Reporting Items for Systematic Reviews and Meta-Analyses (PRISMA) guidelines^17^.

| **Section and Topic** | **Item #** | **Checklist item** | **Location where item is reported** |
| --- | --- | --- | --- |
| **TITLE** | | |  |
| Title | 1 | Identify the report as a systematic review. | p1 |
| **ABSTRACT** | | |  |
| Abstract | 2 | See the PRISMA 2020 for Abstracts checklist. | p4 |
| **INTRODUCTION** | | |  |
| Rationale | 3 | Describe the rationale for the review in the context of existing knowledge. | p6 |
| Objectives | 4 | Provide an explicit statement of the objective(s) or question(s) the review addresses. | p6 |
| **METHODS** | | |  |
| Eligibility criteria | 5 | Specify the inclusion and exclusion criteria for the review and how studies were grouped for the syntheses. | pp7-8 |
| Information sources | 6 | Specify all databases, registers, websites, organisations, reference lists and other sources searched or consulted to identify studies. Specify the date when each source was last searched or consulted. | p7 |
| Search strategy | 7 | Present the full search strategies for all databases, registers and websites, including any filters and limits used. | Appendix 3 |
| Selection process | 8 | Specify the methods used to decide whether a study met the inclusion criteria of the review, including how many reviewers screened each record and each report retrieved, whether they worked independently, and if applicable, details of automation tools used in the process. | p8 |
| Data collection process | 9 | Specify the methods used to collect data from reports, including how many reviewers collected data from each report, whether they worked independently, any processes for obtaining or confirming data from study investigators, and if applicable, details of automation tools used in the process. | p8 |
| Data items | 10a | List and define all outcomes for which data were sought. Specify whether all results that were compatible with each outcome domain in each study were sought (e.g. for all measures, time points, analyses), and if not, the methods used to decide which results to collect. | p8 |
|  | 10b | List and define all other variables for which data were sought (e.g. participant and intervention characteristics, funding sources). Describe any assumptions made about any missing or unclear information. | p10 |
| Study risk of bias assessment | 11 | Specify the methods used to assess risk of bias in the included studies, including details of the tool(s) used, how many reviewers assessed each study and whether they worked independently, and if applicable, details of automation tools used in the process. | p9 |
| Effect measures | 12 | Specify for each outcome the effect measure(s) (e.g. risk ratio, mean difference) used in the synthesis or presentation of results. | SPP p9 |
| Synthesis methods | 13a | Describe the processes used to decide which studies were eligible for each synthesis (e.g. tabulating the study intervention characteristics and comparing against the planned groups for each synthesis (item #5)). | pp9-10 |
|  | 13b | Describe any methods required to prepare the data for presentation or synthesis, such as handling of missing summary statistics, or data conversions. | pp9-10 |
|  | 13c | Describe any methods used to tabulate or visually display results of individual studies and syntheses. | p10 |
|  | 13d | Describe any methods used to synthesize results and provide a rationale for the choice(s). If meta-analysis was performed, describe the model(s), method(s) to identify the presence and extent of statistical heterogeneity, and software package(s) used. | p10 |
|  | 13e | Describe any methods used to explore possible causes of heterogeneity among study results (e.g. subgroup analysis, meta-regression). | p10 |
|  | 13f | Describe any sensitivity analyses conducted to assess robustness of the synthesized results. | p10 |
| Reporting bias assessment | 14 | Describe any methods used to assess risk of bias due to missing results in a synthesis (arising from reporting biases). | p10 |
| Certainty assessment | 15 | Describe any methods used to assess certainty (or confidence) in the body of evidence for an outcome. | p10 |
| **RESULTS** | | |  |
| Study selection | 16a | Describe the results of the search and selection process, from the number of records identified in the search to the number of studies included in the review, ideally using a flow diagram. | p11 |
|  | 16b | Cite studies that might appear to meet the inclusion criteria, but which were excluded, and explain why they were excluded. | p11 |
| Study characteristics | 17 | Cite each included study and present its characteristics. | p12 |
| Risk of bias in studies | 18 | Present assessments of risk of bias for each included study. | pp13-14 |
| Results of individual studies | 19 | For all outcomes, present, for each study: (a) summary statistics for each group (where appropriate) and (b) an effect estimate and its precision (e.g. confidence/credible interval), ideally using structured tables or plots. | p15 |
| Results of syntheses | 20a | For each synthesis, briefly summarise the characteristics and risk of bias among contributing studies. | Appendix 5 |
|  | 20b | Present results of all statistical syntheses conducted. If meta-analysis was done, present for each the summary estimate and its precision (e.g. confidence/credible interval) and measures of statistical heterogeneity. If comparing groups, describe the direction of the effect. | p15 |
|  | 20c | Present results of all investigations of possible causes of heterogeneity among study results. | pp16-19 |
|  | 20d | Present results of all sensitivity analyses conducted to assess the robustness of the synthesized results. | p16 |
| Reporting biases | 21 | Present assessments of risk of bias due to missing results (arising from reporting biases) for each synthesis assessed. | p17 |
| Certainty of evidence | 22 | Present assessments of certainty (or confidence) in the body of evidence for each outcome assessed. | p15 |
| **DISCUSSION** | | |  |
| Discussion | 23a | Provide a general interpretation of the results in the context of other evidence. | p23 |
|  | 23b | Discuss any limitations of the evidence included in the review. | pp20-22 |
|  | 23c | Discuss any limitations of the review processes used. | pp20-23 |
|  | 23d | Discuss implications of the results for practice, policy, and future research. | pp23-26 |
| **OTHER INFORMATION** | | |  |
| Registration and protocol | 24a | Provide registration information for the review, including register name and registration number, or state that the review was not registered. | p7 |
|  | 24b | Indicate where the review protocol can be accessed, or state that a protocol was not prepared. | p7 |
|  | 24c | Describe and explain any amendments to information provided at registration or in the protocol. | pp13-14 |
| Support | 25 | Describe sources of financial or non-financial support for the review, and the role of the funders or sponsors in the review. | p27 |
| Competing interests | 26 | Declare any competing interests of review authors. | p27 |
| Availability of data, code and other materials | 27 | Report which of the following are publicly available and where they can be found: template data collection forms; data extracted from included studies; data used for all analyses; analytic code; any other materials used in the review. | p27 |

*Table 1: PRISMA reporting checklist, adapted from Page et al^17^.*

**Appendix 2: Supplementary Background**

*Confounding by indication*

Observational studies are vulnerable to confounding by indication, whereby ketamine is preferentially selected for haemodynamically unstable patients. Foster et al reported greater mean shock index and over double the incidence of absolute hypotension (SBP <90) in a mixed ED cohort^51^, while Kuza and Van Berkel reported greater severity of injury as well as greater haemodynamic instability trauma populations^23, 26^. Controlling for this effect requires randomisation, infrequently encountered in the literature.

*Ketamine dose*

It was beyond the scope of this review to investigate dose-dependent effects of ketamine on post-induction haemodynamics. Mattson et al found an association between doses below 1.25mg/kg and reduced PIH incidence^44^, replicated in a subgroup of this cohort with SI >0.8^45^. This finding is not universal: a large registry study found no association between increased dose and hypotension incidence or resuscitation requirement^46^. These observational studies are potentially affected by indication bias, whereby lower doses of ketamine are administered to more unstable patients.

**Appendix 3: Search Strategies**

1. PubMed

| #1 | RSI[tiab] OR “Rapid Sequence Induction*”[tiab] OR “Rapid Sequence Intubation*”[tiab] OR “Emergency Intubation*”[tiab] OR “Emergency Induction*”[tiab] OR “Emergency Anaesthe*”[tiab] OR “Emergency Anesthe*”[tiab] OR "Rapid Sequence Induction and Intubation"[Mesh] OR "Rapid Sequence Induction and Intubation/adverse effects"[Mesh] OR "Rapid Sequence Induction and Intubation/methods"[Mesh] OR "Intubation, Intratracheal"[Mesh] OR "Intubation, Intratracheal/adverse effects"[Mesh] OR "Intubation, Intratracheal/methods"[Mesh] |
| --- | --- |
| #2 | Ketamine[tiab] OR Ketamin[tiab] OR Ketofol[tiab] OR "Ketamine"[Mesh] OR "Ketamine/administration and dosage"[Mesh] OR "Ketamine/adverse effects"[Mesh] OR "Ketamine/therapeutic use"[Mesh] OR "Ketamine"[Mesh:NoExp] |
| #3 | “Blood Pressure”[tiab] OR “Blood-Pressure”[tiab] OR BP[tiab] OR SBP[tiab] OR MAP[tiab] OR “Systolic Blood Pressure”[tiab] OR “Systolic Blood-Pressure”[tiab] OR “Mean Arterial Pressure”[tiab] OR Hypotens*[tiab] OR Haemodynamic*[tiab] OR Hemodynamic*[tiab] OR “Post-induction hypotension”[tiab] OR “post induction hypotension”[tiab] OR “postinduction hypotension”[tiab] OR “peri-induction hypotension”[tiab] OR “peri induction hypotension”[tiab] OR “post-intubation hypotension”[tiab] OR “post intubation hypotension”[tiab] OR “postintubation hypotension”[tiab] OR “peri-intubation hypotension”[tiab] OR “peri intubation hypotension”[tiab] OR "Hypotension/chemically induced"[Mesh] OR "Hemodynamics/drug effects"[Mesh] |
| #4 | #1 AND #2 AND #3 |

*Table 2: Full search strategy for PubMed.*

1. Embase

| #1 | RSI:ti,ab OR ‘Rapid Sequence Induction*’:ti,ab OR ‘Rapid Sequence Intubation*’:ti,ab OR ‘Emergency Intubation*’:ti,ab OR ‘Emergency Induction*’:ti,ab OR ‘Emergency Anaesthe*’:ti,ab OR ‘Emergency Anesthe*’:ti,ab OR ‘rapid sequence induction’/exp |
| --- | --- |
| #2 | Ketamine:ti,ab OR Ketamin:ti,ab OR Ketofol:ti,ab OR ‘ketamine’/exp |
| #3 | ‘Blood Pressure’:ti,ab OR BP:ti,ab OR SBP:ti,ab OR MAP:ti,ab OR ‘Systolic Blood Pressure’:ti,ab OR ‘Mean Arterial Pressure’:ti,ab OR Hypotens*:ti,ab OR Haemodynamic*:ti,ab OR Hemodynamic*:ti,ab OR ‘Post-induction hypotension’:ti,ab OR ‘post induction hypotension’:ti,ab OR ‘postinduction hypotension’:ti,ab OR ‘peri-induction hypotension’:ti,ab OR ‘peri induction hypotension’:ti,ab OR ‘post-intubation hypotension’:ti,ab OR ‘post intubation hypotension’:ti,ab OR ‘postintubation hypotension’:ti,ab OR ‘peri-intubation hypotension’:ti,ab OR ‘peri intubation hypotension’:ti,ab OR 'induced hypotension'/exp OR 'hypotension'/de |
| #4 | #1 AND #2 AND #3 |

*Table 3: Full search strategy for EmBase.*

1. Cochrane Library

| #1 | RSI OR “rapid sequence induction” OR “rapid sequence intubation” OR “emergency intubation” OR “emergency induction” |
| --- | --- |
| #2 | emergency NEXT an*sthe* |
| #3 | MeSH rapid sequence induction and intubation (exp) |
| #4 | #1 OR #2 OR #3 |
| #5 | ketamine OR ketofol |
| #6 | MeSH ketamine (exp) |
| #7 | #5 OR #6 |
| #8 | BP OR SBP OR MAP |
| #9 | “blood pressure” OR “systolic blood pressure” OR “mean arterial pressure” |
| #10 | hypotens* OR h*modynamic* |
| #11 | “post induction hypotension” OR “peri induction hypotension” OR “postinduction hypotension” |
| #12 | “post intubation hypotension” OR “peri intubation hypotension” OR “postintubation hypotension” |
| #13 | MeSH hypotension (exp) |
| #14 | #8 OR #9 OR #10 OR #11 OR #12 OR #13 |
| #15 | #4 AND #7 AND #14 |

*Table 4: Full search strategy for the Cochrane Library.*

1. Clinicaltrials.gov

(RSI OR “rapid sequence induction” OR “rapid sequence intubation” OR “emergency induction” OR “emergency intubation” OR “emergency anaesthetic” OR “emergency anaesthesia” OR “emergency anesthetic” OR “emergency anesthesia”) AND (Ketamine OR ketofol) AND (BP OR SBP OR MAP OR “blood pressure” or “systolic blood pressure” OR “mean arterial pressure” OR hypotension OR hypotensive OR haemodynamic OR haemodynamics OR haemodynamically OR hemodynamic OR hemodynamics OR hemodynamically OR “post-induction hypotension” OR “post induction hypotension” OR “postinduction hypotension” OR “peri-induction hypotension” OR “peri induction hypotension” OR “post-intubation hypotension” OR “post intubation hypotension” OR “postintubation hypotension” OR “peri-intubation hypotension” OR “peri intubation hypotension”)

1. Scopus

(TITLE-ABS-KEY(RSI) OR TITLE-ABS-KEY(“rapid sequence induction*”) OR TITLE-ABS-KEY(“rapid sequence intubation*”) OR TITLE-ABS-KEY(“emergency intubation*”) OR TITLE-ABS-KEY(“emergency induction*”) OR TITLE-ABS-KEY(“emergency an*sthe*”)) AND (TITLE-ABS-KEY(Ketamine) OR TITLE-ABS-KEY(Ketofol)) AND (TITLE-ABS-KEY(BP) OR TITLE-ABS-KEY(SBP) OR TITLE-ABS-KEY(MAP) OR TITLE-ABS-KEY(“systolic blood pressure”) OR TITLE-ABS-KEY(“blood pressure”) OR TITLE-ABS-KEY(“mean arterial pressure”) OR TITLE-ABS-KEY(hypotens*) OR TITLE-ABS-KEY(h*modynamic*) OR TITLE-ABS-KEY(“post induction hypotens*”) OR TITLE-ABS-KEY(“post-induction hypotens*”) OR TITLE-ABS-KEY(“postinduction hypotens*”) OR TITLE-ABS-KEY(“peri induction hypotens*”) OR TITLE-ABS-KEY(“peri-induction hypotens*”) OR TITLE-ABS-KEY(“post intubation hypotens*”) OR TITLE-ABS-KEY(“post-intubation hypotens*”) OR TITLE-ABS-KEY(“postintubation hypotens*”) OR TITLE-ABS-KEY(“peri intubation hypotens*”) OR TITLE-ABS-KEY(“peri-intubation hypotens*”))

**Appendix 4: Examples of Rejected Studies**

Selected examples of studies rejected at full-text review stage are discussed below:

Studies presenting only continuous outcome measures of post-induction haemodynamics, without dichotomous incidence data:

- Jabre et al^14^: RCT comparing ketamine versus etomidate in critically ill patients.
- Kadish et al^38^: ED trauma patients induced with ketamine versus historical controls induced with etomidate.

Studies presenting PIH incidence without breaking this down into patients receiving ketamine versus alternatives:

- Levin et al^35^: OCS including haemorrhaging ED trauma patients, comparing PIH incidence when with propofol versus ketamine or etomidate, with the latter group not further subdivided.
- Mohr et al^36^: OCS measuring various exposures, including induction agent choice, and incidence of various adverse events, including PIH, but not breaking down PIH incidence by agent choice.
- Stassen et al^37^: similar to Mohr et al.

**Appendix 5: Critical Appraisal of Eligible Studies**

1. *RCTs included in sensitivity analyses iii) and iv)*

Ali et al^15^

- RSI in OR for source control surgery in septic shock patients (well-matched baseline lactate) requiring continuous noradrenaline infusion.
- Ketamine and fentanyl groups both received 0.05mg/kg midazolam as co-induction, isofluorane to maintain anaesthesia.
- Pre-induction: all patients received 30ml/kg fluid + further boluses until non-responsive.
- Post-induction ventilated to 6ml/kg tidal volumes (no airway pressure data).

Srivilaithon^33^

- ED patients with suspected sepsis.
- Well-matched baseline characteristics between groups.
- Both received single induction agent (no co-induction drugs).
- PIH (a secondary outcome) definition did not specify a time frame.
- Higher rate of rocuronium administration (vs suxamethonium) in ketamine group.

Knack et al^34^

- Excluded patients if potentially hazardous to raise HR/BP (e.g. suspected intracranial hypertension), and female patients without negative pregnancy test.
- Some crossover: 8 patients (including 7 in etomidate group) received ketamine pre-hospital.
- PIH a secondary outcome.

Matchett et al^12^

- RSI by dedicated “Airway team”.
- Well-matched baseline characteristics.
- Applied Vanderbilt definition of cardiovascular collapse^51,52^ (a secondary outcome).

Elsherbiny et al^32^

- Sepsis-3 definition of septic shock, although only 62% on continuous noradrenaline pre-induction (54% in ketamine group, 69% in thiopental group).
- RSI in OR for unspecified surgical procedures.
- All resuscitated with 30ml/kg fluid, plus additional boluses until non-responsive (in which case commenced on noradrenaline infusion targeting MAP 65).

1. *RCTs excluded from sensitivity analyses iii) and iv)*

Nakajima et al^21^

- Abstract only.
- Alternate-day randomisation (odd days ketamine, even days etomidate).
- Describes well-matched groups without presenting data.

1. *OCSs included in sensitivity analysis iv)*

Zuin et al^16^

- STEMI patients, excluding SBP <90, pre-RSI inotropic support, or pre-hospital intubation.
- Groups well matched for pre-induction haemodynamics and ACS features (including peak troponin concentration).
- Average time to PIH: 4 minutes in ketamine group, 7 in midazolam group.

Kang et al^29^

- Abstract only.
- PIH definition not specified.
- Adjusted OR after controlling for concurrent medications including co-induction agents and vasopressors).
- Significantly reduced PIH incidence with ketamine in overall cohort and non-sepsis subgroup (not broken down further); no significant difference in sepsis subgroup.

Kunkel et al^54^

- Pre-hospital RSI, mostly by flight paramedics.
- Confounding by indication: much more haemodynamically unstable ketamine cohort vs etomidate. Corrected for to some extent by PIH definition (applied differently to patients depending on pre-induction BP).

Ishimaru et al^28^

- ED RSI in patients with shock index ≥0.9.
- Multivariable and propensity-matched analyses performed for adjusted ORs; former used in meta-analysis.
- Pre-induction haemodynamic not elaborated on, but “medical shock” significantly more common indication for RSI in ketamine group (vs respiratory failure, trauma, or other).

Grant et al^41^

- PIH definition specified “new hypotension” which could only be met by patients with SBP <90. Extrapolation from presented data required to calculate denominators.
- Patients receiving very low doses of each medication were excluded from the meta-analysis.
- By stratifying data into those receiving versus not receiving each drug, no-drug intubations were likely included in the meta-analysis as part of the comparator group.

Tangkulpanich et al^31^

- Study designed to identify risk factors for PIH; it is likely that there was crossover between induction agents.

Hsieh et al^27^

- Abstract only.
- Adjusted OR from logistic regression analysis.

Nakornchai et al^30^

- Conducted in non-trauma ED.
- Study designed to identify risk factors for PIH; it is likely that there was crossover between induction agents.

Bakhsh et al^43^

- Convenience sample (patients recruited when principal investigator was on shift).
- Study supplementary materials detail RSI protocol: optional pre-medication with fentanyl (1-3μg/kg at administrator’s discretion), etomidate 0.3mg/kg in normotensive patients with option to replace this with 1-2mg/kg ketamine in hypotensive patients.
- RSI protocol also dictates 2-minutely boluses of adrenaline or phenylephrine to maintain SBP ≥100.
- Unclear to what extent this was followed: pre-induction haemodynamics between groups were well-matched despite this study excluding patients requiring bolus vasopressors.

Stanke et al^13^

- Pre-hospital RSI.
- No significant difference in PIH incidence with ketamine vs etomidate, including within subgroups divided by haemodynamic stability or age.
- Subgroup analysis included indications of respiratory failure and altered mental status within the “medical subgroup”, excluding 9% of non-trauma patients (for whom relevant data was not available).

King et al^17^

- Pre-hospital RSI in patients with ROSC following cardiac arrest from non-traumatic cardiac arrest.
- Distinct PIH definitions for patients with and without baseline SBP <90.
- Incidence and dose of fentanyl administration was comparable between groups.

Mattson et al^44^

- Abstract only.
- Compared PIH incidence when administering reduced doses of each agent, finding a significantly reduced incidence when administering ≤1.25mg/kg ketamine vs >1.25mg/kg.
- A subgroup analysis for those with SI>0.9 was presented in a separate abstract but contained insufficient data to include in the subgroup analysis^45^.

Foster et al^51^

- Confounding by indication present: ketamine group more haemodynamically unstable at baseline. No correction to PIH definition to account for this.
- Authors acknowledge that they were unable to exclude patients with pre-existing haemodynamic instability due to disproportionate effect on sample size of ketamine group.

Kim et al^42^

- Multiple definitions of PIH depending on pre-induction haemodynamics.
- Logistic regression applied to some outcomes but not to PIH incidence with ketamine vs all alternatives; unadjusted frequency data used for meta-analysis.
- Study also conducted analyses into effect of drug dosage on PIH incidence; no significant difference was found between different ketamine doses.

Driver et al^46^

- NEAR registry data.
- Multivariable analysis did not find association between increased dosage of ketamine or etomidate and PIH incidence; only raw (unadjusted) data was available for PIH incidence overall.
- Sensitivity analyses excluding patients with pre-existing hypotension and including only patients with pre-existing shock also did not identify association between induction agent dose and PIH incidence.

Lyon et al^11^

- Comparison of “3:2:1” (3μg/kg fentanyl, 2mg/kg ketamine, 1mg/kg rocuronium) vs etomidate with suxamethonium in pre-hospital trauma patients.
- Both study arms had option (at administrator’s discretion) to reduce dose (1:1:1 or 0.15m/kg etomidate) or to omit induction agents altogether in haemodynamic compromise.
- Of the 8 instances of δSBP/MAP >20% in the ketamine group, all were given full dose, and only 1 had absolute hypotension (SBP <90); the single instance of δSBP >20% in the etomidate group was given a reduced dose and suffered absolute hypotension.
- Etomidate group (receiving no fentanyl) was associated with significantly higher rates of post-induction hypertension.

1. *OCSs excluded from sensitivity analysis iv)*

Van Berkel et al^26^

- PIH definition extended to 24 hours, therefore all patients not surviving to 24 hours were excluded from analysis.
- Statistically significant confounders: greater exposure to benzodiazepines post-RSI in ketamine group; greater opioid co-induction rate in etomidate group; ketamine group less haemodynamically stable at baseline.
- Adjusted OR with propensity-matched analysis (in which no significant difference between groups in pre-induction BP or opiate co-administration).

Breindahl et al^22^

- Greater incidence of shock pre-intubation in ketamine group, and lower median baseline blood pressure.
- PIH definition: vasopressor use (no threshold for use specified).
- The above combine to result in significant potential for bias.

Kuza et al^23^

- Included trauma patients undergoing RSI within 24h of admission (including in OR).
- Only short-term PIH outcome available (in keeping with other studies) was vasopressor requirement within 15 minutes (no threshold for use specified); remainder were set to 24h.
- Ketamine group was both less haemodynamically stable and more severely injured; in combination with PIH being defined by vasopressor requirement, this results in significant potential for bias.

Price et al^24^

- Incidence and dose of fentanyl administration was comparable between groups.
- Measured incidence of “new hypotension”: SBP <90 or MAP <60 where these were not present pre-induction. However, these data are presented over a denominator of the entire cohort (including those with pre-induction hypotension).
- It is not possible to calculate the numbers of patients with pre-induction hypotension from the presented data, which states that this was present in 7.3% of the 50 patients administered ketamine 9.1% of the 50 patients administered etomidate.
- Given the low overall numbers (n=100), the discrepancies above have potential to alter the study’s result.

Pollack et al^25^

- PIH not defined.
- Methods describe fentanyl as an optional analgesic to be given alongside induction agents. However, the results section classifies fentanyl as an alternative induction agent.
- The study quotes n =7466, with ketamine n= 3463, etomidate n = 1381, midazolam n = 889, fentanyl n = 1381. However, as fentanyl was an optional co-induction drug, it is likely this refers to 7466 drug administrations (not all for the purpose of inducing anaesthesia) in an unknown number of patients. There is therefore significant crossover between groups and doubt over the correct denominator values.

**Appendix 6: Data Extraction for Subgroup Analyses**

Table 5 demonstrates the data extracted for each subgroup analysis.

| **Subgroup** | **Studies Included** | **Studies Discarded** | **Total n** | **Ketamine** | **Comparator** |
| --- | --- | --- | --- | --- | --- |
| **Overall** | 27 | 0 | 31956 | 8472 | 23484 |
| **Comparator** | 26 | 1 | 30977 | 8156 | 22821 |
| **Stability** | 10 | 17 | 16683 | 2731 | 13952 |
| **Population** | 14 | 13 | 18525 | 3260 | 15265 |
| **Setting** | 26 | 1 | 31408 | 8244 | 23164 |

*Table 5: numbers of studies & participants included in each subgroup analysis.*
